# Exploring cultural, social, and biological factors influencing obesity onset in two racial-ethnic groups in Quibdó, Colombia

**DOI:** 10.1017/jns.2024.44

**Published:** 2024-10-17

**Authors:** Paula Andrea Castro-Prieto, Daniela Molano-Moreno, Diego I. Lucumí

**Affiliations:** 1 Universitat Autònoma de Barcelona-Departament de Geografía & Centre d’Estudis Demogràfics-CERCA, Barcelona, Spain; 2 School of Medicine, University of the Andes, Bogotá, Colombia; 3 School of Government, University of the Andes, Bogotá, Colombia

**Keywords:** Colombia, Ethnicity, Obesity, Race

## Abstract

Obesity rates in Colombia are increasing, with variations among racial and ethnic groups. Studies on adult obesity often address socio-economic status, gender, and education but neglect racial-ethnic influences, notably in areas like Quibdó. Therefore, based on the theory of triadic influence, we conducted a qualitative study to identify biobehavioural, social, and cultural phenomena that, from the perspectives of the participants, influence the onset of obesity in Afro-Colombian and indigenous in Quibdó in 2022. The stratification variables were race, ethnicity (Afro-Colombian and Indigenous), and educational level (secondary or higher). Based on a literature review of qualitative studies that commonly explored food culture, nutritional status, and physical activity in analysing obesity within racial and ethnic populations, we incorporated these categories into our research methodology through semi-structured interviews. A framework analysis was used as a qualitative methodology to organise and analyse the collected data. We conducted 21 semi-structured interviews, 13 with the Afro-Colombian population and eight with indigenous inhabitants. The results indicate that cultural beliefs, forced displacement/migration, and alterations in public order have resulted in changes in food security, food culture, and physical activity practices, affecting the onset of obesity. Notably, distinctions in cultural beliefs regarding food culture and health as factors influencing obesity were observed between Afro-Colombians and the Indigenous populations; however, educational differences within the same racial ethnic group were not predominant. Findings indicate obesity is influenced by cultural, social, and biobehavioural factors, especially in regions with racial-ethnic communities facing complex conditions, necessitating targeted racial-ethnic public health policies.

## Introduction

Obesity is a risk factor for chronic conditions such as type 2 diabetes, high blood pressure, and coronary heart disease.^(1–3)^ Obesity is a significant public health problem owing to its high incidence and economic burden.^(4)^ Prevalence rates are increasing in Latin America, including Colombia. For instance, in Mexico, the prevalence reached 29.9% in 2006^(5)^ to 36.1% in 2019,^(6)^ and in Brazil, it was from 8.6% in 2006 to 14.6% in 2019.^(7)^ In Colombia, a quasi-cohort analysis derived from nutritional surveys (ENSIN, acronym in Spanish) conducted in 2005, 2010, and 2015 revealed an increase of 6.1 percentage points between 2005 and 2015, reaching 21.3% in this last year.^(8)^

Evidence from studies in Colombian population suggests that diverse factors influence obesity. In cities such as Cali, low socioeconomic status (SES) is correlated with higher obesity rates in women than in those with medium/high SES.^(9)^ Similarly, in Medellín, low SES, education, and income below 1,400,000 Colombian pesos were associated with obesity, particularly among women.^(10)^ These findings align with previous research demonstrating an association between obesity and sex, age, education, occupation, and income. Women face an elevated risk, which increases with age and educational level.^(11)^ However, ethnicity and race remain underexplored in obesity-related research.

Afro-Colombians and Indigenous people, the two main racial-ethnic groups in Colombia, represent together approximately 13% of the population.^(12)^ They often hold distinct customs and beliefs^(13)^ and have historically faced social inequalities such as limited land ownership, lower income^(14,15)^ and unequal access to healthcare services.^(16)^ Geographically, these groups have resided in areas in which armed conflict^(17)^ has led to forced displacement and alterations in their dietary habits. Consequently, shifts towards more calorie-dense foods, such as tubers, soups, sausages, flour, and panela water (sugar type), to satisfy their hunger have been observed.^(18)^ Additionally, both indigenous and Afro-Colombian households reported higher levels of food insecurity (77% and 68.9%, respectively) than the general population (52.3%).^(19)^ In 2015, 26% of Afro-Colombians, were obese and the prevalence among indigenous population was 18.8%.^(8)^ All these facts suggest of need a deeper understanding of obesity as a public health issues in these racial-ethnic groups.

Existing studies on obesity in Colombia often adopt a quantitative approach and include a race and ethnicity^(20,21)^ but typically do not delve into the first-hand perspectives of obese individuals and do not investigate the factors and influences contributing to the onset of this condition. Moreover, modifiable behaviours such as diet and physical activity, have also been understudied from a racial-ethnic perspective in Colombia.^(22)^

Therefore, a qualitative study is needed to understand factors associated with the onset of adult obesity, especially in two racial-ethnic communities that cohabite in the same territory, such as Quibdó. To understand obesity from a racial-ethnic perspective, we incorporated the Triadic Influence Theory, which provides higher-order descriptions and explanations of health-related behaviours. It proposes three streams of influence: the cultural environment, social background, and biology/personality. Cultural environment is related to attitudes, including religion and ethnicity. Social background relates to normative beliefs such as family systems, parenting styles, social attachment, and normative social beliefs. Biology and personality are related to self-efficacy, self-esteem, self-control, and self-assessment including biological resilience, character, and social skills.^(23)^ The Triadic Influence Theory has also been used by other researchers in nutrition and public health.^(24–28)^

Our study aimed to analyse cultural, social, and individual factors that influence the development of obesity in Afro-Colombian and Indigenous in Quibdó in 2022 by using the Triadic Influence Theory. We considered this theory is appropriate to guide this study because it provides a comprehensive perspective on diverse issues involving behavioural, environmental, and social factors that have been defined as precursors to obesity.^(29)^

## Materials and methods

### Study design and site

Between July and November 2022, we conducted a qualitative descriptive study in Quibdó.

We chose Quibdó, the capital of the department of Chocó in the Colombian Pacific region (CPR), because most of its population is recognised as Afro-Colombian, with a small proportion of indigenous people.^(30)^ We established possible pathways of influence based on the existing food and nutritional literature.^(31–37)^ In our analysis, cultural influences encompassed phenomena related to food culture,^(22)^ whereas social influences were related to food security and physical activity.^(22,38)^ In terms of biology/personality influences, we connected themes to beliefs and perceptions of weight and healthy and unhealthy habits, as these may be mediated or influenced by personality aspects, such as self-esteem, self-control, and self-assessment, as suggested by theory and literature.^(23,32–34)^ We also included additional themes that emerged during the analysis.

According to population projections, for 2022 Quibdó had 139,740 inhabitants,^(39)^ constituting 26.4% of the population of Chocó and is composed of 92.8% Afro-Colombians and 4.0% Indigenous people. In socioeconomic terms, 72.8% of the population lived with unsatisfied basic needs, and 11.6% were in conditions of misery.^(40)^ Furthermore, in the department of Chocó, there have been educational inequalities, e.g. the enrolment rate in the population aged 5–16 years old was 84%, while in Bogotá, the country’s capital, this was 96.4%.^(41)^

Regarding the nutritional situation, it bears to note that in 2015, around 21% of adults in the CPR were obese, higher than the national average of 18.7%.^(19)^

### Sampling and data collection

We carried out a stratified purposeful sampling^(42)^ to capture the main variations in the factors that influence the onset and persistence of obesity in Quibdó.^(43)^ The variables selected for stratification were race-ethnicity and educational level. Race-ethnicity was categorized as Afro-Colombians and indigenous, and educational attainment was categorized as low (incomplete secondary or lower) or high (secondary or higher) (Table 1). Categories were determined based on the educational indicators in the Chocó Department. According to the 2018 Census, the illiteracy rate exceeded 20%, while the net coverage of secondary education reached 51.09%, compared to 70.84% in the country’s capital. Consequently, access to higher education in these departments is challenging (Table 1).^(44)^

**Table 1.** Stratified purposeful sampling

Race/ethnicity	Educational attainment low	Educational attainment higher
Afro-Colombian	6	6
Indigenous	6	6
Total	12	12

Inclusion criteria for the study were being an adult (18 years of age or over) who identify as Afro-Colombian or member of an indigenous community, resident in Quibdó, with a body mass index >30 Kg/m^2^ or waist >80 cm in women and >90 cm in men.^(45)^

Participants were identified by researchers of the Nursing Department of Universidad Tecnológica del Chocó, who knew community leaders or associations due to their work in public health in Quibdó. Before data collection, all the participants signed an informed consent form.

A semi-structured interview guide was used to gain insights into people’s thoughts regarding obesity as outlined in the theoretical framework.^(46)^ The interview guides were developed based on the primary findings of a previous literature review.^(31–37)^ The main topics included food culture, dietary memories, and dishes for special celebrations. Themes, such as food availability, access, and acquisition methods, which constitute the concept of food security, were identified within the social domain. Additionally, barriers to and facilitators for physical activity were addressed.^(22,38)^ In the biology/personality domain, perceptions of health and disease, beliefs about weight, views on healthy and unhealthy habits, preferred foods and dishes, and necessary and unnecessary foods were explored.^(23,32–34)^ Semi-structured interviews allowed us to understand the selected topics while also providing flexibility to include new themes or emerging categories based on the development of each interview.^(46)^ Prior to the interviews, we piloted the guide with four Colombians volunteers. The questions, phrasing, language, and sequences were reviewed and revised during the pilot process. In addition, the average application time was evaluated and adjusted as necessary. The average time for each interview was 45 minutes, during which information was collected (Supplementary Material 1).

### Data analysis

The interviews and their analyses were conducted by two health professionals under the guidance, support, and feedback of a senior qualitative researcher. It is worth noting that the interviews were conducted in Spanish, the Colombia official language.

Concerning the analysis, based on triadic influence theory and its influences (cultural environment, social background, and biology/personality), we used the five steps of qualitative framework analysis comprising five distinct stages: familiarisation, identification of a thematic framework, indexing, charting, mapping, and interpretation.^(47,48)^ The first step was to listen to the tape, read the notes and transcripts verbatim, and define the main ideas. The identification stage recognises the emerging themes and issues in the data. At this point, priority themes and issues emerged and were intended to allow the data to guide the identification of themes and topics. The third step involved identifying specific themes in the collected data. It is important to note that the two researchers reviewed the four interviews simultaneously to observe whether the final interpretations were similar. We discussed this analysis and found similar findings; therefore, we decided to divide the interviews, which comprised the entire sample of 21 participants. The abstraction and synthesis of information are developed during charting. Finally, the mapping and interpretation entailed an examination of the main aspects outlined in the charts. Simultaneously, we translated the data into English to construct charts and maps. The final step involved the use of charts and maps to analyse the nature of the phenomenon and explain the relationships between the findings.^(47,48)^ *Atlas.ti 9* was used to manage the data, including the indexing and charting.

### Ethics approval and consent to participate

This study was conducted according to the guidelines laid down in the Declaration of Helsinki and all procedures involving human subjects/patients were approved by the Comitè d’Ètica en la Recerca of the Universitat Autònoma de Barcelona (CEEAH 6074). Written informed consent was obtained from all the subjects. The databases created for this study were anonymised for analysis, results, and conclusions.

## Results

The general characteristics of the participants are presented in Table 2. We collected information from 21 of the 24 participants we selected in the sampling process, including 13 Afro-Colombians and eight indigenous individuals. It is important to note that four indigenous people were not interviewed because of prior commitments, resulting in their unavailability to participate. Additionally, we encountered limitations in accessing specific neighbourhoods where some Indigenous people lived. Despite these challenges, we believe that information saturation was achieved during the data collection, as indicated by the results.

**Table 2.** Characteristics of the study participants

Variables	Afro Colombians	Indigenous
Age group		
20–29	2	2
30–39	4	2
40–49	4	2
50–62	3	0
Unknown	0	2
Sex		
Men	6	3
Women	7	5
Educational attainment		
Low	6	3
Higher	7	5
**Total**	13	8

Qualitative data analysis identified three streams of influence as possible explanations for the appearance and persistence of obesity. Specifically, the participants mentioned elements of food culture as well as social, biological, and personality influences that were possibly associated with obesity. The roles of these three influences are presented in the following subsections, considering participants’ perceptions and connections (Table 3).

**Table 3.** Factors that influence adult obesity in Quibdó, according to study participants

Influence	Categories	Emergent categories	Afro-Colombian	Indigenous
Food culture				
	Dietary memories		Bush meat, fish, soups during childhood	
	Soul food		Rice with coconut, atollado, sancocho, envueltos, hojaldras, and arepas	Fish cooked after fishing
		Religion and cultural beliefs	▪ Easter: Dish of papaya or coconut ▪ San Pacho Festival: rice with all, sausage, pork, crackling, sancocho, and high-fat meat snacks	▪ Daily product: Chicha ▪ Menarche: atollado
Social				
	Availability and access to food products		▪ AHE: native fruits such as chontaduro, borojo, árbol de pan, and guanabana ▪ ALE & IHE: home-produced foods ▪ Carbohydrates: Potatoes, yuca, rice, legumes, and banana ▪ Proteins: river fish, bush meat, chicken, eggs, and processed meat	
	Food acquisition methods		▪ Market square ▪ Supermarket chains ▪ Local shops ▪ Shopping centre ▪ ALE: home-produced foods such as coriander, garlic, onion, plantain, and yuca	
	Barriers to food access		▪ Scarcity and price increase due to transport disruptions caused by demonstrations and strikes ▪ Erosion ▪ Subienda ends	
	Barriers and facilitators for practicing physical activity		▪ The lack of security by the presence of criminal organisations ▪ Lack of space and motivation ▪ Prices of gym memberships	
		Alterations of the public order	▪ Demonstrations and strikes	
		Migration/forced displacement	▪ Forced displacement	
Biology/personality				
	Perceptions of health and disease		▪ AHE: – Health: Physical and emotional wellbeing and healthy diet – Disease: Discomfort, exhaustion, lack of health, worries, and unrest ▪ ALE: – Health: All of qualities of being active – Disease: Sadness, discomfort, and sickness	▪ IHE: – Health: Wellbeing, good relationships, harmony with nature, and self-esteem – Disease: Pain and bodily alterations due to environmental, psychological, and emotional factors ▪ ILE: – Health: Absence of pain – Disease: Presence of pathologies
	Healthy things		▪ AHE: Physical and playful activities and nutritious diet ▪ ALE: home produced foods	▪ ILE: cheerful positive
		Religion and cultural beliefs: healthy things	ALE: connection with God	
	Diet		Balance of food intake, which allows for the development of daily routines	
	Ideal diet		Portion sizes	
	Healthy foods		Fish, chicken, legumes, meat, fruits, and vegetables	Vegetables: onions and tomatoes
	Unhealthy foods		Pork crackling and sausages, fast food, mercury in fish, and sedentary lifestyles	
	Favourite dishes		River fish	
	Beliefs about weight		▪ AHE: – Underweight individuals might have parasites, and food intake – Overweight: overeating, chronic conditions ▪ ALE: – Underweight people are not strong enough – Overweight: having conditions	IHE: – Overweight: diseases such as hypercholesterolaemia, diabetes, and sedentary lifestyles
		Using traditional plants and medicines	Due to the lack of confidence in the health system	

*AHE, highly educated Afro-Colombians; **ALE,** low-educated Afro-Colombians; **IHE,** highly-educated indigenous people; **ILE,** low-educated indigenous people.

### Food culture influences

Participants identified different areas that could influence the cultural stream. Among the areas identified were dietary memories such as child feeding, learning to cook, soul food, and how religious beliefs might influence diet and the cultural sphere. It is important to note that no differences were found in educational attainment among the racial and ethnic communities.

#### Afro-Colombian population

Concerning dietary memories, which may relate to intake patterns in adulthood, Afro-Colombian respondents agreed that bush meat, fish, and soups were their main meals during childhood. In addition, parents, especially mothers, learned cooking during childhood. Another notable dietary memory is the significance of soul food. For instance, the main soul foods of Afro-Colombians are cereals, roots, tubers, and plantains. Additionally, rice with coconut, atollado^1^, sancocho^2^, envueltos^3^, hojaldras^4^, and arepas^5^, sometimes prepared with the products of their harvest, is considered soul food for Afro-Colombians. These meals typically have high carbohydrate content.

[“The rice with coconut… It was delicious and a habit. Now, I am an adult and I yearn for coconuts because I grew up in Urabá, Antioquia, so I went to the courtyard, got the coconuts I wanted, and made rice my way. Here, if I want to make a pound of rice with coconut, the price of the coconut is defined by its weight, but it could be small. I have stopped making it because you buy a coconut for 6000 or 8000 pesos, and if you cut it and find that is not good, to whom I will complain… The seller and you are not to blame, can you imagine, this is what I miss” (ALE4)]

[“Of course, when we were children, my mother used to prepare many of what we called envueltos chispeados. We would harvest corn, grind it, and she would cook these chispeados for us. She used to cook us colleanos, with a bottle for breakfast. Since we produced chocolate, she would grind it and serve it to us in the morning with cheese. It was a very delicious meal and, at that time, very healthy. Very healthy because of the corn. We also harvested rice and a lot of bananas, so we had daily meals like that” (AHE1)]

Religious beliefs play a significant role in cultural influence. Afro-Colombians stated that religious festivals have promoted the preparation of unique recipes over the years. For instance, on Easter, they made a dish of papaya or coconut and bread made only with egg yolk, and avoided meat intake. Easter week coincides with the month when the availability of fish in different CPR rivers increases, known as “La Subienda”. Consequently, people have easier access to fish during this period, but availability varies significantly due to price changes during the remainder of the year.

Another example is the San Pacho Festival. This festival is held annually in September to commemorate Saint Francis of Assisi. In addition to the extraordinary folkloric and cultural events in Quibdó, there is an increase in commonly consumed dishes, such as rice with all^6^, sausage, pork crackling, sancocho, and high-fat meat snacks, which contain high levels of carbohydrates and saturated fat, and sodium. These dishes are fried in oil and accompanied by alcoholic beverages.

#### Indigenous population

Indigenous people include a traditional drink called “chicha”, a refreshing beverage, as a part of their food culture. Chicha is prepared by the fermentation of products such as corn, sugarcane, or panela (products based on conventional sugars), which, after fermentation, are converted into alcoholic drinks. Chicha is not considered a problematic beverage, because it is part of their food culture. Additionally, chichas with low fermentation levels are also offered to children daily.

[“No, chicha is natural and normal since my mother prepared it when I was a child. They always made their chicha with a sweet taste for the children and another with more fermentation for the adults” (IHE5)]

[“We eat at home what our parents accustomed us to eat, yes, because we eat a little salad, and we drink a little juice because for us the custom is chicha” (IHE7)]

Concerning dietary memories, indigenous respondents agreed that bush meat, fish, and soups were their main meals during childhood. In addition, highly educated indigenous people (IHE) said that they moved from their villages to Quibdó when they were young or due to forced displacement; therefore, the Afro-Colombians taught them to cook instead of their indigenous families living in their places of origin. Another dietary memory is the soul food. Fish cooked after fishing was mentioned as a soul food by the indigenous people, who insisted that they changed their nutritional patterns due to migration.

Regarding religious beliefs as part of cultural streams, most indigenous people mention menarche as an important community festival. In this celebration, indigenous people paint their bodies, wear typical costumes, play the flute, dance, and connect with nature to celebrate the beginning of womanhood. In addition, they also prepare “atollado”, or rice with different meats and plantain, and chicha of panela due to the high sugarcane prices in Quibdó. The interviewees mentioned that the honouree might not eat much because she would have a large stomach, which is frowned upon, suggesting that obesity influences this cultural practice. This belief confirms that overeating is a known risk factor for obesity; however, they do not refer to it for the entire population but only for women. At the same time, they pointed out that they do not often celebrate festivities such as San Pacho, because it is an Afro-Colombian tradition.

[“When a young lady becomes a Miss, she cannot eat all kinds of food. She will eat grilled plantains and fish in small portions. According to belief, if she eats a lot, she might have a big belly. So, they eat a little, a little water too” (IH1)]

### Social influences

The interviewees discussed influences such as the availability and access to specific food products, methods of acquiring food, barriers, and facilitators of food access within a social context. Additionally, they addressed obstacles and facilitators to engaging in physical activity, disruptions to public order, and experiences of migration and forced displacement.

There were some distinctions in educational attainment levels. For example, highly educated Afro-Colombians (AHE) recognise native fruits in their region. Less-educated Afro-Colombians (ALE) mentioned home-produced foods, a sentiment echoed by the indigenous population (IHE). However, overall, the differences between racial-ethnic groups were minimal; therefore, subsequent results do not include racial and ethnic divisions.

As mentioned by all respondents, food products recognised as available were often high in carbohydrates, such as potatoes, yuca, cereals (mainly rice), legumes, and bananas. Natural proteins such as river fish, bush meat, chicken, eggs, and processed meat are also acknowledged. The AHE predominantly mentioned native fruits, such as chontaduro, borojo, árbol de pan, and guanabana, although these were noted as challenging to access compared with other areas on CPR.

[“The diet is based on things that chocoano likes to eat. For instance, plantain, meat, fish, chicken, eggs and specific fruits that are produced in Chocó such as borojó, árbol de pan, caimito” (AHE1)]

Regarding food acquisition methods, interviewees from Afro-Colombian and Indigenous people indicated similar primary locations, including the market square, supermarket chains, local shops, and unique shopping centres. Additionally, ALE mentioned home-produced foods, such as coriander, garlic, onion, plantain, and yuca.

Barriers to food access for both Afro-Colombian groups and indigenous people include scarcity and price increases due to transport disruptions caused by demonstrations and strikes. Other factors restricting access were cultural customs erosion, which led to processed food substitutions and declining soil fertility, affecting banana supply. Furthermore, the consumption of river fish declined after the end of the La Subienda season, affecting dietary habits.

[“Here, in Quibdó, the truth is that when demonstrations and strikes happen, sometimes it is challenging to find legumes, such as onions or potatoes, besides the price rises. On the other hand, plantain and fish are easy to find because they are brought to the river” (IHE4)]

[“For instance, rice, arepa, and potatoes are the cheapest, but plantain, yuca, primitivo and banana are scarce; maybe they are not grown. Today, a ration of plantain costs 120000 pesos, and I cannot afford it. For example, when we lived in our municipality, we could get fruit, such as maracuyá, orange, guamo and avocado, which we cultivated”(ILE3)]

Both Afro-Colombian and indigenous groups stressed the importance of consuming certain food groups within their cultural contexts. However, they recognised that access and availability are influenced by social factors, especially forced displacement and alterations in the public order, which impact dietary choices.

Regarding physical activity, participants from both groups engaged in sports during childhood but not adulthood, indicating a trend of decreased activity in later life. The lack of security, mainly due to the presence of criminal organisations, hinders outdoor activities. As mentioned by the AHE and ALE, additional barriers, such as time restrictions and lack of space and motivation, further impede physical activity. Furthermore, the AHE and ALE discussed private gymnasiums.

[“A shootout happens here between different gang leaders. Someone is saying, I want to run this and this neighbourhood and I cannot anymore. I was playing with the children when I heard it, and I told them, “‘Let’s go, kids, back home” because you never know when a stray bullet might kill a child, and then I would be responsible because I am playing with the children as a teacher. That is my responsibility”(ILE6)]

[“I am afraid to run alone on the streets because people are being killed and kidnapped today; then, I am scared to run at five in the morning, and I cannot find anyone to accompany me” (ILE1)]

Despite the availability of public space facilitating physical activity, such as the Aeroparque, Malecón, and basketball and volleyball pitches within neighbourhoods, inconveniences persisted. These included household activities, obligations, work schedules, and the distance between homes and “El Aeroparque”, the city’s primary location for sports practice.

[“The truth is that there are many places, if you want to go the Aeroparque, malecón, shopping malls, even some hotels promote the physical activity. Nowadays, the parks in Quibdó are being equipped with exercise machines. In the past, they were only provided with things for the children, such as swings. So, if you do not want to go to the city centre, you can go to these spaces. There is a park near my house, and I go there to practice physical activity, so everyone has the chance to practice. The reason people do not practice physical activity is that they do not want to do it, but everybody has the capacity to do it at home, neighbourhood, in shopping centres and Aeroparque” (AHE8)]

[“Sometimes yes, for example, in the mornings I cannot because I work and, in the evenings, I go home quickly because I cannot stay late at night” (ALE12)]

Additionally, participants were generally unaware of government-led initiatives promoting physical activity.

### Biology-personality influence

Personality was identified as the primary domain of this study. Participants mentioned issues related to their nutritional status. Topics included perceptions of health, illness, health and unhealthy things, beliefs about weight, views on healthy and unhealthy habits, preferred food and dishes, necessary and unnecessary foods, and the use of traditional plants and medicines in case of illness. Regarding this influence, educated individuals in both Afro-Colombian and indigenous communities mentioned obesity as a risk factor for the onset of non-communicable diseases. Furthermore, differences in perceptions of health and disease were identified between racial and ethnic groups.

#### Afro-Colombian population

Regarding perceptions of health and illness, the AHE mentioned a concept that links physical and emotional well-being with a healthy diet, and they defined illness as ailments, discomfort, exhaustion, lack of health, worries, and unrest. In contrast, ALE related health to all the qualities of being active, such as vitality, developing activities, being productive, and being connected with God, and defined illness as feelings of sadness, discomfort, and sickness.

[“Healthy situations are feeling good and without problems. Sometimes, I have issues that stress me out, but I do not suffer because I am so connected to God. I like the Christian life, so it keeps me healthy, and for every problem I have, God will find a solution. I am not saying I do not have problems, but I know that God from heaven is the resolution to all the difficulties. When I have a God in my heart, I do not think about problems” (ALE6)]

Regarding healthy and unhealthy things, the AHE mentioned physical and playful activities and nutritious diet. Simultaneously, both the AHE and ALE perceive diet as a balance of food intake, which allows for the development of daily routines and is mediated by culture. Additionally, the AHE stated that it was time to share information with others. In their perceptions of an ideal diet, both the AHE and ALE emphasised the significance of portion sizes, highlighting the importance of consuming large portions. They also regarded fatty foods such as pork crackling and sausages, fast food, mercury in fish, and sedentary lifestyles as unhealthy.

Furthermore, they suggested a list of foods grouped by macronutrients such as proteins, fats, and carbohydrates. In addition, they mentioned some healthy foods such as fish, chicken, legumes, meat, fruits, and vegetables. Regarding dishes preferred by food groups, both the AHE and ALE agreed that their favourite dish was river fish, while seafood was another protein preferred by the ALE. Furthermore, beans were the favourite of AHE in the legume group, while ALE liked them the least. When asked about their favourite dish, there was no mention of healthy food groups, such as vegetables or fruits.

[“The diet is based on things that chocuano likes to eat. For instance, plantain, meat, fish, chicken, eggs, and specific fruits that are produced in Chocó such as borojó, árbol de pan, caimito” (AHE1)]

Despite the elements provided to understand their ideal diet, they do not align with their actual dietary habits, as salty and fatty foods are accepted and consumed according to the findings of the cultural stream. The ALE also indicated that home-produced foods, living in connection with God, and playful activities were considered healthy, whereas chemicals in food were classified as harmful.

Regarding weight beliefs, the AHE believes that underweight individuals might have parasites and inadequate food intake. Conversely, overweight was associated with overeating, increased body mass index, and chronic conditions. This confirmed that the participants acknowledged a connection between obesity and the onset of chronic diseases. The ALE mentioned that underweight people are not strong enough and referred to the overweight population as having conditions; however, in the past, the weight of people was accepted without judgment.

Finally, some interviewees stated that due to a lack of confidence in the health system, they use plants as their families taught them when they were children and believe in God as a mechanism to treat specific pain.

[“Before, I used to be a fat person. Now, I have lost weight, but I feel pain in the soles of my feet, and I have not gone to the doctor because they prescribe ibuprofen or paracetamol. Besides, the tests are good, and they suggest I go somewhere else; I arrive almost dead. So, I said I do not have to waste my time there. I prefer to prepare whatever with my plants at home because when I feel pain in my kidneys, I get plants called the desbaratadora, Santamaría, tres dedos and riñonera. After I cooked this water and the pain disappeared, I asked myself Why I went to the doctor if Chocó had a defective health system. Health here is linked to Christ, held in the hands of God. We do not have a health system” (ALE4)]

#### Indigenous population

The IHE expressed that health involves well-being, good relationships, harmony with nature, and self-esteem, as opposed to pain and bodily alterations due to environmental, psychological, and emotional factors, which are accompanied by imbalances in the perception of illness. It is important to note that low-educated Indigenous people (ILE) consider health to be the absence of pain, and disease to be the presence of pathologies.

[“For me, health is life, and the Embera world means to feel good, dress well, have good friendships, harmony with nature, and everything. The illness is imbalance; if a human body is sick, it no longer has that love for the other to share, so illness leads to the disappearance of a human being because this is the reason for the extinction of the world in the future if people keep getting sick, people are going to die” (IHE5)]

Regarding healthy and unhealthy things, the ILE mentioned that being cheerful is positive, but bad feelings and lack of hygiene are considered unhealthy. On the other hand, the IHE said that their diet only consisted of food from local farmers, providing them with natural foods such as plantains, bananas, yuca, rice, and fish, and pointed out that milk was unnecessary for the body.

Concerning preferred dishes by food groups, in the protein source group, both IHE and ILE respondents agreed that their favourite dish was river fish. Beans were the least liked food in the legume group. Regarding vegetables, despite being recognised as healthy foods, only the ILE stated that they liked them, but only referred to onions and tomatoes, and no other vegetables. Furthermore, none of the participants mentioned fruits.

Regarding weight beliefs, statements by the IHE added specific diseases related to being overweight, such as hypercholesterolaemia, diabetes, and sedentary lifestyles, and considered childhood overweight to be well regarded.

## Discussion

Obesity is a significant risk factor for cardiometabolic diseases^(49)^ and has been widely studied in the medical field. Although obesity has been related to social determinants of health,^(11,50–54)^ racial-ethnic approaches in low- and middle-income countries, such as Colombia, are scarce. Using triadic influence theory and seeking to contribute to this field, we examined the factors that influence the development of obesity in racial-ethnic groups in a middle-sized Colombian population. Our findings show that dietary memories such as child feeding, learning to cook, soul food, and religious beliefs define food culture. Access and availability, which are integral components of food security dimensions, are facilitators and barriers within the social sphere. At the same time, healthy and unhealthy things, preferred foods and dishes, necessary and unnecessary foods, and beliefs about weight were linked to personality influences. Furthermore, alterations in public order, migration, and forced displacement were the emergent categories most relevant to this study. Adding these three streams and emergent types gave qualitative explanations about the onset of obesity in Afro-Colombian and indigenous people who cohabit in Chocó’s capital city, Quibdó.

In terms of *food cultural* influence, in racial-ethnic groups, food is a means of transmission and preservation of their cultures and social cohesion, especially in migration contexts, as in the case of the ancestral community La Playa Renaciente in Cali, Colombia, where the Afro-Colombian population predominantly lived.^(55)^ Simultaneously, one study supported the idea that alcohol consumption among indigenous people in Colombia is a social construct as it involves dynamic systems and authorities, transculturation, and interculturality.^(56)^ Despite the importance of food culture, the consumption of highly saturated foods, as reported by the Afro-Colombian population, is a risk factor for obesity.^(57)^ On the other hand, in our study, the indigenous population do not consider “chicha” harmful to their health, which is dangerous when ingested in excess, and the literature coincides with alcohol as a risk factor for obesity^(58)^ and cardiometabolic risk.^(59,60)^

Regarding *social influences*, changes in food access and availability may alter intake patterns and contribute to obesity.^(22)^ In Quibdó, these two axes are affected by social conditions such as demonstrations and strikes. Interviewers reported that roads were frequently closed during public order disturbances, leading to price rises and the scarcity of essential products, such as onions and legumes. A similar situation was reported in a study in Brazil, where the strikes of general truck drivers were associated with reduced food availability and rising prices.^(61)^ According to evidence, it is important to recognise that food insecurity may increase the likelihood of obesity in this type of contexts.^(62)^

Furthermore, forced displacement and migration in Colombia have driven significant social changes, with many municipalities experiencing population losses and others becoming receiver sites. For example, in 2002, nearly 90% of Colombia’s municipalities were population expellers, with some losing more than half their residents, such as Bojayá (94.7%) and Riosucio (76.1%). Quibdó, capital of the department of Chocó, was particularly affected, becoming a receptor site.^(63)^ This shift affects local food chains and security, potentially leading to changes in eating patterns that contribute to obesity.^(64–66)^ Studies across Colombia have revealed dietary shifts towards high-calorie, low-nutrient foods among displaced populations. For instance, in Santander (a Colombian department), the families of victims of forced displacement relied on high-calorie, high-sugar, and high-carbohydrate products.^(67)^ Similarly, research in Bogotá observed a decrease in protein intake,^(18)^ whereas in Putumayo (another Colombian department), displaced people often sold eggs to afford cheaper, lower-quality food, thereby increasing the risk of obesity.^(68)^ Addressing food insecurity and displacement-related issues is essential for mitigating this trend.

Participants also reported low levels of physical activity due to several factors, including the lack of outdoor spaces, sidewalks, and signage near their homes, and the predominant use of motorcycles for transportation. Concerns about theft due to criminal gangs in Quibdó further deterred physical activity. These findings indicate that social conditions and urban infrastructure contribute to reduced physical activity, increasing the risk of obesity in Quibdó. One explanation is that migration from rural to urban areas in Latin America and the Caribbean has exacerbated urban infrastructure challenges, affecting housing and transportation.^(69)^ Many places in Quibdó may be classified as similar to a slum due to inadequate access to water, sanitation, and infrastructure, as well as high levels of overcrowding and security issues,^(70)^ which limit physical activity and promote a sedentary lifestyle linked to obesity. Access to green spaces,^(71)^ parks, and public transportation^(72)^ are associated with increased physical activity, but racial-ethnic minority groups, like those in Quibdó, often face barriers to accessing these amenities.^(73)^

The above explanations show that Quibdó represents an environment where individuals may experience prolonged exposure to social adversity across the lifespan, increasing their risk of obesity onset. Factors such as low social class, subjective social status, education, poverty, and living in deprived areas contribute to uncertainty about parental employment and accommodation, which can occur in Quibdó. These conditions may lead to chronic stress and psychological strain, triggering obesity-related behaviours, such as smoking and drinking, with consequences for the offspring.^(74)^ The stress response activates the hypothalamic-pituitary-adrenal (HPA) axis, releasing cortisol, which promotes fat storage and central obesity.^(75)^ Future research should explore variations in social stressors across cities where an important segment of racial-ethnic groups in Latin America live, and their impact on obesity risk.

In the *personality* sphere, Afro-Colombians and Indigenous people mentioned that a healthy diet includes fish, chicken, legumes, meat, fruits, and vegetables. However, individuals often adhere to beliefs and customs regarding food, which may lead them to consume foods that they recognise as unhealthy. Furthermore, Afro-Colombian interviewees recognised the importance of health and disease control for themselves and their communities. Nevertheless, in situations of illness, they preferred to appeal to traditional medicine because of lack of confidence and difficulties in accessing the health system. This is similar to Amazonas who live in an indigenous community; they indicated that they prefer not to go to the hospital due to the lack of timely care, and that they always recommend the same things.^(76)^ Another critical point is that for highly educated people in both racial-ethnic groups, obesity is a risk factor for cardiometabolic diseases, which suggests that participants with higher educational attainment have more access to health information.^(77)^ However, it was evident that, despite knowing about this topic, the prevention strategies that they were implementing were low.

These factors provide a vital reflection on how public policies intervene in obesity prevention from a racial-ethnic perspective in Colombia. On one hand, a lack of confidence in the health system creates distance between racial-ethnic communities and primary care providers, hindering access to some of the interventions proposed by evidence, including counselling focused on diet, physical activity, and behaviour change.^(78)^ On the other hand, this study highlights the lack of strategies and programs to prevent obesity in Colombia, which, besides implementation, require check-ups and a longer time frame because, such interventions require at least nine months to observe changes in dietary patterns and physical activity as mechanisms to reduce or prevent obesity, as mentioned by Mastellos *et al.* in 2014.^(79)^

### Strengths and limitations

A critical strength of the study is that it involved two racial-ethnic groups living in the same city but with different customs and beliefs, despite sharing similar social situations. Additionally, to our knowledge, it constitutes the first qualitative study to understand the obesity phenomenon in Quibdó, Colombia. However, this study had some limitations. Because we performed the last step of the analysis in English, we may not have captured the real essence or feelings of the participants. Nevertheless, our cited translations were reviewed by two researchers born in CPR who guided us in trying to show the essence of the interviewers’ contributions. Furthermore, three indigenous people with low education levels who did not attend the interviews may have contributed to other cultural, social, and biology/personality influences. However, the themes among the indigenous people who participated in the study were recurrent, suggesting that the research identified the main issues of the phenomena under investigation.

### Conclusion

The nutritional status of being obese in Quibdó is influenced by food culture as well as social and personality streams, which are shaped by racial-ethnic groups more so than educational attainment. However, a common theme across these factors is the impact of cultural beliefs, forced displacement, disruptions to public order, and lack of confidence in the health system, which catalyse changes in access, availability, and dietary and physical activity patterns, thus affecting the onset of obesity. Therefore, our findings offer new insights into obesity trends from a racial-ethnic perspective, particularly in communities affected by complex social conditions, making this study a pioneering effort in Colombia. Additionally, the study provides valuable insights into the food and nutritional behaviours of racial-ethnic communities, especially in the absence of current nutritional data, given the recent government’s implementation of measures such as food labelling as one of the ways to prevent risk factors such as obesity and non-communicable diseases.

## Supporting information

Castro-Prieto et al. supplementary material Castro-Prieto et al. supplementary material

## Footnotes

1 Rice with smoke meat, salad cheese, scallion, tomatoes, and cumin.

2 Soup with smoked chicken, potatoes, yuca, plantain, and smoke cheese.

3 Corn-based product topped with corn or plantain leaves

4 Wheat flour-based fried food

5 Typical food from Colombia that it prepared with pre-cooked white corn meal, cheese and butter

6 Rice with sausage, salty cheese, onions, tomatoes, garlic, pepper, and artificial flavouring

## Abbreviations

**CPR:** Colombian Pacific Region; **AHE:** highly-educated Afro-Colombians; **ALE:** low-educated Afro-Colombians; **IHE:** highly-educated indigenous people; **ILE:** low-educated indigenous people.

## Supplementary material

The supplementary material for this article can be found at https://doi.org/10.1017/jns.2024.44
