# Supplementary material for: Exploring cultural, social, and biological factors influencing obesity onset in two racial-ethnic groups in Quibdó, Colombia
Source: J Nutr Sci. 2024 Oct 17;13:e65. doi: 10.1017/jns.2024.44 (PMC11503850; doi:10.1017/jns.2024.44)
Supplement: Castro-Prieto et al. supplementary material [file S2048679024000442sup001.docx]

**Supplementary material: Interview guide**

**Cultural: food culture**

- When do you gather with family, friends, or the community to eat? What dishes do you usually eat? Describe them.
- What foods and preparations did you eat when you were a child and young? Do you still consume them, yes/no, and why?
- Do you enjoy cooking? Who taught you? What preparations have lasted throughout your life?
- At what age did you learn to cook? Who did you learn? Do children and young people currently learn to cook at that age?
- Do you miss any preparation that you no longer eat regularly?
- In what situations do you prepare "special" meals? What are these "special" preparations?
- Are there any celebrations in your territory that involve the consumption of special dishes?

**Social: food security**

- What foods are available in your territory?
- How often do you acquire food?
- What is the method of obtaining food?
- Where do you usually get food (bought, gifted, home-produced foods)?
- What foods do you usually obtain (bought, gifted, home-produced foods)?
- Which foods are easy to obtain?
- Which foods are difficult to obtain?
- How do you consider the price of food in your territory?

**Social: physical activity**

- For you, what does physical activity/exercise mean?
- Do you engage in physical activity/exercise? How often? Where do you usually engage in physical activity/exercise?
- In your neighbourhood/city, are there spaces for engaging in physical activity/exercise?
- What facilities does your neighbourhood/city have for engaging in physical activity/exercise?
- What difficulties do you encounter in your neighbourhood/city when it comes to engaging in physical activity/exercise?
- In your neighbourhood/city, are you familiar with and/or enrolled in any programs for engaging in physical activity/exercise?

**Biology/personality: nutritional and health situations**

- What is health to you? What is illness?
- What activities, things, or situations do you consider healthy? What are not healthy?
- What does food mean to you?
- How should an ideal diet be?
- What procedures do you consider when preparing food?
- What are your favourite foods and preparations? Why?
- What foods and preparations do you dislike? Why?
- Which foods do you consider necessary for the body?
- Which foods do you not consider necessary for the body?
- What knowledge, beliefs, and traditions do you know about:
- Diseases related to body weight?
- What is excess weight to you? What is obesity?
- What was the perception of weight in your community when you were a child/young?
